# The transposable element-derived transcript of LIN28B has a placental origin and is not specific to tumours

**DOI:** 10.1007/s00438-023-02033-1

**Published:** 2023-06-03

**Authors:** Chiemi F. Lynch-Sutherland, Lorissa I. McDougall, Peter A. Stockwell, Suzan N. Almomani, Robert J. Weeks, Jackie L. Ludgate, Teena K. J. B. Gamage, Aniruddha Chatterjee, Joanna L. James, Michael R. Eccles, Erin C. Macaulay

**Affiliations:** 1 Department of Pathology, Dunedin School of Medicine, University of Otago, Dunedin, 9054 New Zealand; 2 Department of Physiology, Faculty of Medical and Health Sciences, The University of Auckland, Auckland, New Zealand; 3 Department of Obstetrics and Gynaecology, Faculty of Medical and Health Sciences, The University of Auckland, Auckland, New Zealand; 4 Maurice Wilkins Centre for Molecular Biodiscovery, Level 2, 3A Symonds Street, Auckland, New Zealand

**Keywords:** Transposable Elements (TEs), Onco-exaptation, LIN28B, Placental Development

## Abstract

Transposable elements (TEs) are genetic elements that have evolved as crucial regulators of human development and cancer, functioning as both genes and regulatory elements. When TEs become dysregulated in cancer cells, they can serve as alternate promoters to activate oncogenes, a process known as onco-exaptation. This study aimed to explore the expression and epigenetic regulation of onco-exaptation events in early human developmental tissues. We discovered co-expression of some TEs and oncogenes in human embryonic stem cells and first trimester and term placental tissues. Previous studies identified onco-exaptation events in various cancer types, including an AluJb SINE element–*LIN28B* interaction in lung cancer cells, and showed that the TE-derived *LIN28B* transcript is associated with poor patient prognosis in hepatocellular carcinoma. This study further characterized the AluJb–*LIN28B* transcript and confirmed that its expression is restricted to the placenta. Targeted DNA methylation analysis revealed differential methylation of the two *LIN28B* promoters between placenta and healthy somatic tissues, indicating that some TE–oncogene interactions are not cancer-specific but arise from the epigenetic reactivation of developmental TE-derived regulatory events. In conclusion, our findings provide evidence that some TE–oncogene interactions are not limited to cancer and may originate from the epigenetic reactivation of TE-derived regulatory events that are involved in early development. These insights broaden our understanding of the role of TEs in gene regulation and suggest the potential importance of targeting TEs in cancer therapy beyond their conventional use as cancer-specific markers.

**Supplementary Information:**

The online version contains supplementary material available at 10.1007/s00438-023-02033-1.

## Introduction

Transposable elements (TEs) are a class of repetitive DNA sequences that have the ability to move or replicate within the genome (Bourque et al. 2018). Initially, TEs were considered “junk DNA” with no functional role in gene expression or regulation. However, studies have revealed that TEs are involved in regulating gene expression during development and disease, including cancer (Faulkner et al. 2009; Burns 2017; Gerdes et al. 2016). In cancer, dysregulated TEs can function as alternate promoters to activate oncogenes, driving the process of onco-exaptation, which is a common mechanism for oncogene activation in many cancer types (Babaian et al. 2016; Jang et al. 2019). Identifying these TE-derived regulatory events is revealing new mechanisms of oncogenesis and has led to the exploration of TEs as potential therapeutic targets. However, much of the research on TEs and cancer has focused on the identification of cancer-specific TEs, and the potential involvement of TEs in early development and normal physiology remains poorly understood. In this study, we sought to explore the expression and epigenetic regulation of onco-exaptation events in early developmental tissues to broaden our understanding of the role of TEs in healthy human development and cancer.

TEs have significantly contributed to rapidly evolving gene regulatory networks during mammalian evolution (Feschotte 2008; Senft and Macfarlan 2021). This is likely due to the existence of regulatory motifs within TEs, which may have facilitated the co-option of these regions into host gene regulatory networks (Kunarso et al. 2010). Recruitment of TE sequences to function as *bona fide* genes and regulatory elements has been termed ‘exaptation’ (Cornelis et al. 2015). Genes and regulatory elements that contain transposable element sequences can also be referred to as transposable element-derived and transposable element-regulated genes (Lynch-Sutherland et al. 2020). TEs can function as regulators of nearby genes (in *cis*) and can also influence expression of distant genes (in *trans*). There are now documented examples of TEs that function as protein-coding genes, long non-coding (lnc) RNAs, promoters, enhancers, insulators and boundary elements for topologically associated domains (Kelley and Rinn 2012; Chuong et al. 2017; Hadjiargyrou and Delihas 2013; Wang et al. 2014; Zhang et al. 2019).

Some of the first exaptation events were identified in the placenta (Cornelis et al. 2015). Perhaps the earliest example of TE-exaptation in the placenta was the discovery that envelope proteins (syncytins) function to enable the essential fusion of trophoblast cells during placentation (Cornelis et al. 2015; Roberts et al. 2021). TEs have also been implicated in driving tissue specific expression of lncRNAs in the placenta. Specifically, L1PA2, LINE-1 elements appear to function as placental-specific promoters for lncRNAs (Chishima et al. 2018). The placenta is known to have lower levels of DNA methylation than adult somatic tissues, which has likely enabled by the unique methylation landscape of the placenta and may have facilitated the recruitment of somatically silenced TEs (Ng et al. 2010; Reiss et al. 2007). Chuong et al. investigated placental-specific enhancers in rat and mouse trophoblasts (placental epithelial cells) and found that these elements were highly enriched for endogenous retroviral (ERV) sequences, and that retroviral recruitment was enriched in tissue types with lower levels of DNA methylation (Chuong et al. 2013). Further work has demonstrated that ERV-derived enhancer elements are mediated by GATA 2/3 and MSX2 in trophoblast cells and have a role in both repression and activation of trophoblast gene regulatory networks (Branco et al. 2022; Du et al. 2023). Altogether, this suggests that lower levels of DNA methylation at some TE loci may have facilitated the recruitment of normally silenced TEs, thus enabling the evolution and diversification of new regulatory networks in the placenta. Furthermore, many TEs are dynamically regulated throughout early human development and functional TEs tend to be highly cell and stage specifically expressed.

Cancer cells share unique functional characteristics with tissues of early human development (hESCs and the placenta). Replicative immortality, increased proliferative capacity and a distinct metabolism are fundamental stem cell traits that can also be observed in cancer (Afify and Seno 2019; Martello and Smith 2014; Menendez and Alarco´n 2014). Studies have investigated the differentiation status of tumours in relation to invasion and metastasis with fascinating results. It is apparent that expression of differentiation markers declines as a cancer progresses and stem cell markers become more predominant (Liu et al. 2013; Nguyen et al. 2012; Yamada et al. 2014). Intriguingly, some cancers have been shown to express not just early developmental genes derived from the embryo but also those that are exclusive to the extra-embryonic lineage (which gives rise to the placenta). This is notable, because the placenta possesses unique functional properties that are exclusive to placentation and are not seen in any other healthy somatic tissues. These stem from its essential roles in both the nourishment and immunological disguise of the fetus during pregnancy. The human placenta shares striking similarities with cancer cells (Ferretti et al. 2007; Novakovic and Saffery 2013). During the first trimester of pregnancy, the trophoblast cells of the placenta invade into the uterine wall to seek out a blood supply for the developing fetus. Similarly, cancer cells also invade into the surrounding tissue to seek out a bloody supply and sustain growth (Davies et al. 2016; Gude et al. 2004). Moreover, both tissues exhibit manipulation of the host immune system to prevent recognition and immunological rejection. Fascinatingly, there is evidence to suggest that species that have evolved to allow extensive invasion of the placenta show a higher incidence of epithelial cancers (Bronchud 2018). Currently, it remains unknown to what extent the molecular basis of these functional properties is shared between the placenta and cancer; however, some cancers show enriched expression of placental genes. The mechanism by which placental genes become reactivated in cancer is currently unknown, although it seems feasible that dedifferentiation of tumour cells may play a key role in facilitating this, through enabling increased phenotypic plasticity.

Recent literature has revealed a role for TEs in promoting expression of oncogenes in human cancers, a process termed onco-exaptation (Babaian and Mager 2016). Onco-exaptation involves the epigenetic reactivation of TE-derived promoters or enhancers to drive expression of oncogenes, thus promoting oncogenesis. Importantly, the somatically silenced status of regulatory TEs has led to the proposal that these TE–oncogene regulatory relationships are novel to cancer (Babaian et al. 2016). This was based on investigation of the corresponding somatic tissue to the tumour, where the TE-derived promoter was methylated. However, cancers cells can reacquire an epigenetic landscape that is reminiscent of early human developmental tissues (embryonic stem cells and the placenta), and subsequently reactivate early developmental genes (Liu et al. 2013; Rousseaux et al. 2013; Smith et al. 2017; Wang et al. 2013). To this end, the fundamental regulators of pluripotency are all potent oncogenes, suggesting that cancers can repurpose developmental genes to drive malignancy (Patra 2020; Tatetsu et al. 2016; Wang et al. 2013). Based on this idea, we proposed that some early developmental TE-derived genes may become reactivated in cancer, and therefore, some onco-exaptation events may not be novel to cancer. We, therefore, hypothesised that some onco-exaptation events occur because of epigenetic reactivation of TE–gene regulatory relationships that exist in early human developmental tissues and that these may enable cancers to recapitulate some of the properties of early developmental tissues (Lynch-Sutherland et al. 2020).

*LIN-28 Homolog B (LIN28B)* is a well-characterised protein-coding gene that plays a critical role in early development, particularly in the placenta (Ali et al. 2020b; Lozoya et al. 2014). Both *LIN28A* and *LIN28B* are expressed during embryogenesis but are silenced in most healthy somatic tissues. *LIN28B* is an important regulator of placental growth and development and is highly expressed in the human placental tissues, with a 1300-fold upregulation of *LIN28B* mRNA in comparison with *LIN28A* in term placental samples reported (Ali et al. 2020a). Other work has shown upregulation of *LIN28B* in invasive extravillous trophoblasts from first trimester placental samples in comparison with the non-invasive villous trophoblasts (Canfield et al. 2019). Both genes are also expressed in various cancers and are well-described oncogenes (Wang et al. 2015; Zhang et al. 2015; Zhou et al. 2013). Work in mouse models suggests that activation of *LIN28B* alone is sufficient to initiate tumour formation (Nguyen et al. 2014). Guo et al. identified a transcript of *LIN28B* that they described as tumour specific, and their work also demonstrated a crucial role for this transcript in promoting tumour progression of hepatocellular carcinoma (Guo et al. 2018). Further work by Jang et al. discovered that this alternate transcript is promoted within an AluJB SINE element (a type of TE). This work also demonstrated a dominant role for the AluJB SINE element in promoting expression of *LIN28B* in numerous different cancer types. Moreover, they were able to show that deletion of the AluJB element obliterated expression of *LIN28B* in a lung cancer cell line, and that DNA methylation was a crucial mediator of promotor activity (Jang et al. 2019). Both studies report high methylation and no expression of *LIN28B* in the corresponding somatic tissue (Guo et al. 2018; Jang et al. 2019).

Our study profiles onco-exaptation events in both hESCs and placental tissues to evaluate whether they have developmental origins. We discovered that a number of the TE–oncogene interactions identified by Jang et al. are expressed in either embryonic stem cells or the placenta. Notably, we also report that the previously described tumour-specific, TE-derived transcript of *LIN28B* is expressed in human placental tissues and is, therefore, not tumour specific. Our work shows expression of the AluJB SINE element in RNA-Sequencing (RNA-Seq) data generated from placental tissues (samples from both first trimester and term) and an absence of expression in a cohort of eight healthy adult somatic tissues (brain, heart, kidney, lung, liver, ovary, testis and melanocyte). The RNA-Seq analysis was validated by reverse-transcriptase, quantitative polymerase chain reaction (RT-qPCR) to further confirm expression of the TE-transcript in the placenta. Furthermore, targeted deep bisulfite sequencing of both promotor regions of *LIN28B* revealed lower levels of DNA methylation of the SINE promotor in comparison with the canonical promotor in the placenta, while the reverse pattern was observed in the healthy somatic tissues surveyed. Overall, our data supports that the TE-transcript of *LIN28B* is not cancer-specific as it is also expressed in the human placenta. This work in combination with the previous studies on the TE-transcript of *LIN28B* demonstrates the potential for reactivation of placental TE-derived transcripts in cancer. These findings hold potential to be leveraged for the development of new therapeutic strategies.

## Results

### RNA-Seq analysis of previously identified onco-exaptation candidates in developmental tissues

Prior work by Jang et al. identified onco-exaptation by utilising 15 different cancer types from The Cancer Genome Atlas (TCGA) and developing a pipeline to identify TE-derived oncogene transcripts that were highly tumour enriched. They started with a list of 702 oncogenes and analysed 7769 tumour samples and 625 tumour matched normal samples. Their analysis identified a total of 625 TE–oncogene chimeric transcripts, and from these, 129 high-confidence tumour enriched onco-exaptation events were called. Intriguingly, at least one onco-exaptation event was identified in 49.7% of tumours, and the same TE–oncogene interaction arose on average 51 times and often ranged across multiple cancer types. The top ten most prevalent onco-exaptation events were characterised, further revealing that the TE-derived promoter was driving the majority of total oncogene expression, in some cases over 90%. Additional analysis showed that over half of these onco-exaptation events were associated with worse patient prognosis in at least one cancer type (Jang et al. 2019).

Here, the expression of onco-exaptation candidates identified by Jang et al. was investigated using both newly generated RNA-Seq data from 15 first trimester placental lysates and 1 hESC line, and publicly available RNA-Seq data sets from 16 healthy term placental lysates and a further 7 hESC lines. First, a gene transfer format (GTF) file containing annotations for the TEs from onco-exaptation events identified by Jang et al. was created. Expression of the TEs in early developmental RNA-Seq data sets was then quantified using FeatureCounts (Liao et al. 2014). The data was analysed to identify TEs that showed enriched expression in either hESCs or placental tissue lysates (first trimester and term) in comparison with the eight healthy somatic tissues for which RNA-Seq data was available.

**Fig. 1:**
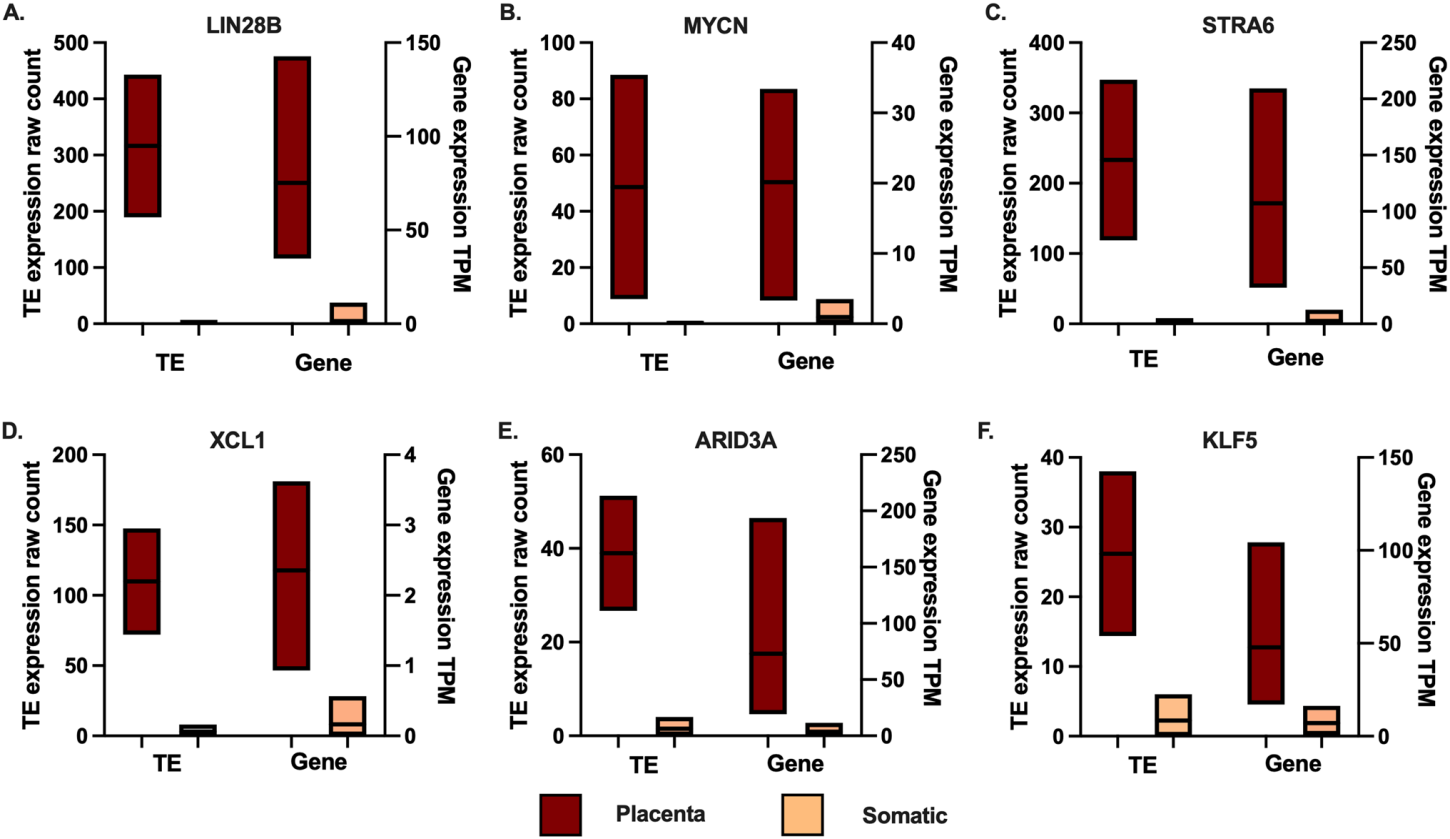
Expression of six onco-exaptation candidates in placental and somatic tissue. TE expression is plotted on the left *Y* axis and gene expression is plotted on the right axis. Placenta *n* = 30, somatic *n* = 8 (brain, heart, kidney, liver, lung, ovary, testis and melanocyte)

This analysis revealed that 75 onco-exaptation TEs showed placental-enriched expression. Placental-enriched TEs were defined as those that had an average expression of over 25 (raw count) in all placental samples and a fold change of more than two in placenta when compared to the average expression across the eight healthy somatic tissues analysed. Of the 129 TE–oncogene interactions identified as being tumour enriched by the Jang et al. analysis, 16 of these TEs showed placental-enriched expression and demonstrated co-expression of the corresponding oncogene in placenta. Expression of these candidates is listed in Supplementary Table 1. Expression of both the TE and corresponding oncogene in placenta and somatic tissues plotted for six onco-exaptation candidates in Fig. 1.

**Fig. 2:**
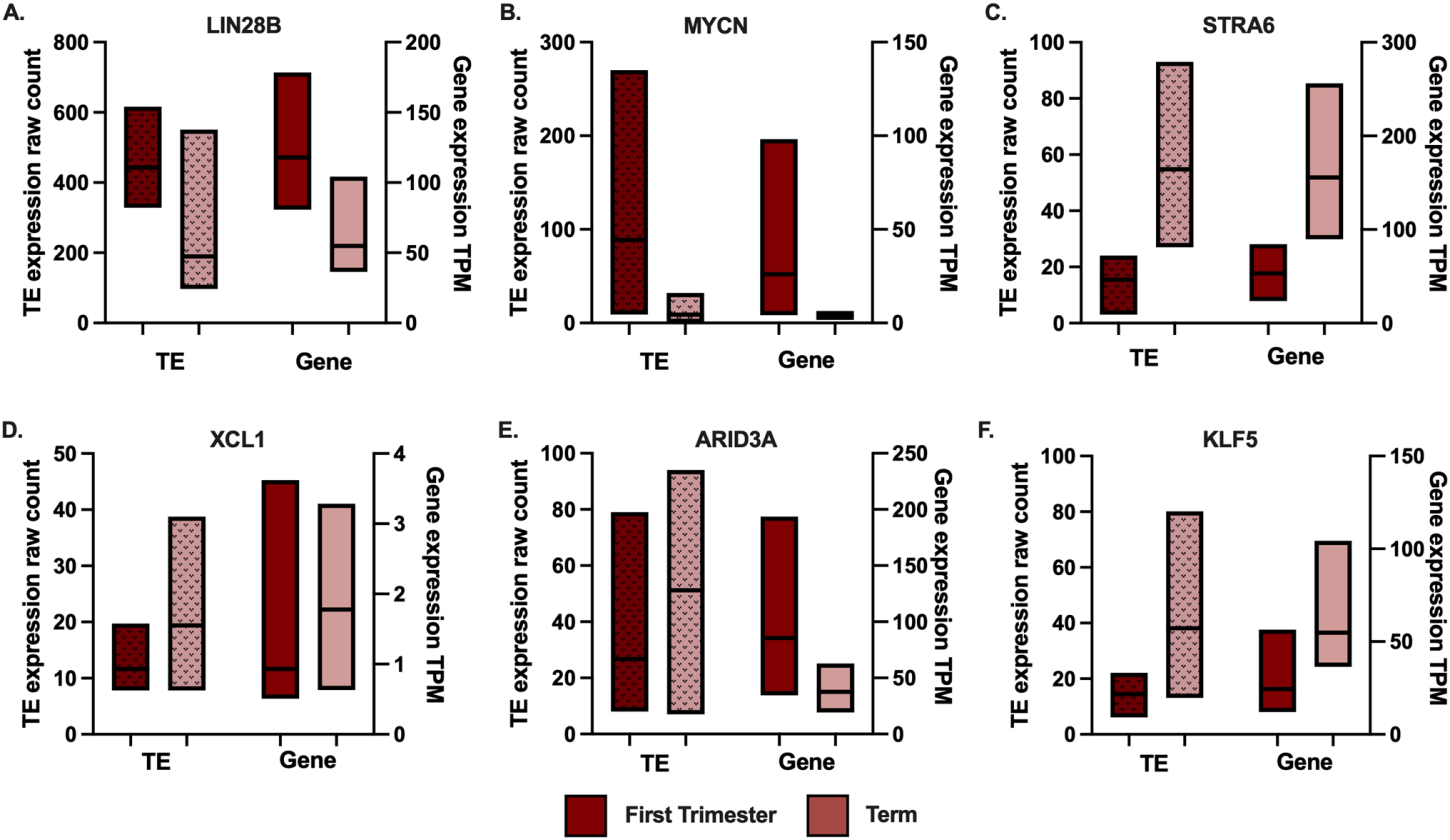
Expression of onco-exaptation candidates in first trimester and term placental tissue. TE expression is plotted on the left *Y* axis and gene expression is plotted on the right axis. First trimester placenta *n* = 14, term placenta *n* = 16

Both the TE and the oncogene show placental-enriched expression for all onco-exaptation events plotted, suggesting that the TE–oncogene regulatory relationships may have function in the placenta. To further investigate whether the level of TE expression corresponded with the level of expression of the corresponding oncogene in placenta, the TE and oncogene expression were plotted in both first trimester and term placental tissues. Expression of the TE and oncogene show a consistent trend across placental gestation for most genes. Specifically, when the TE is up- or down-regulated across gestation the gene appears to show the same trend (Fig. 2).

**Fig. 3:**
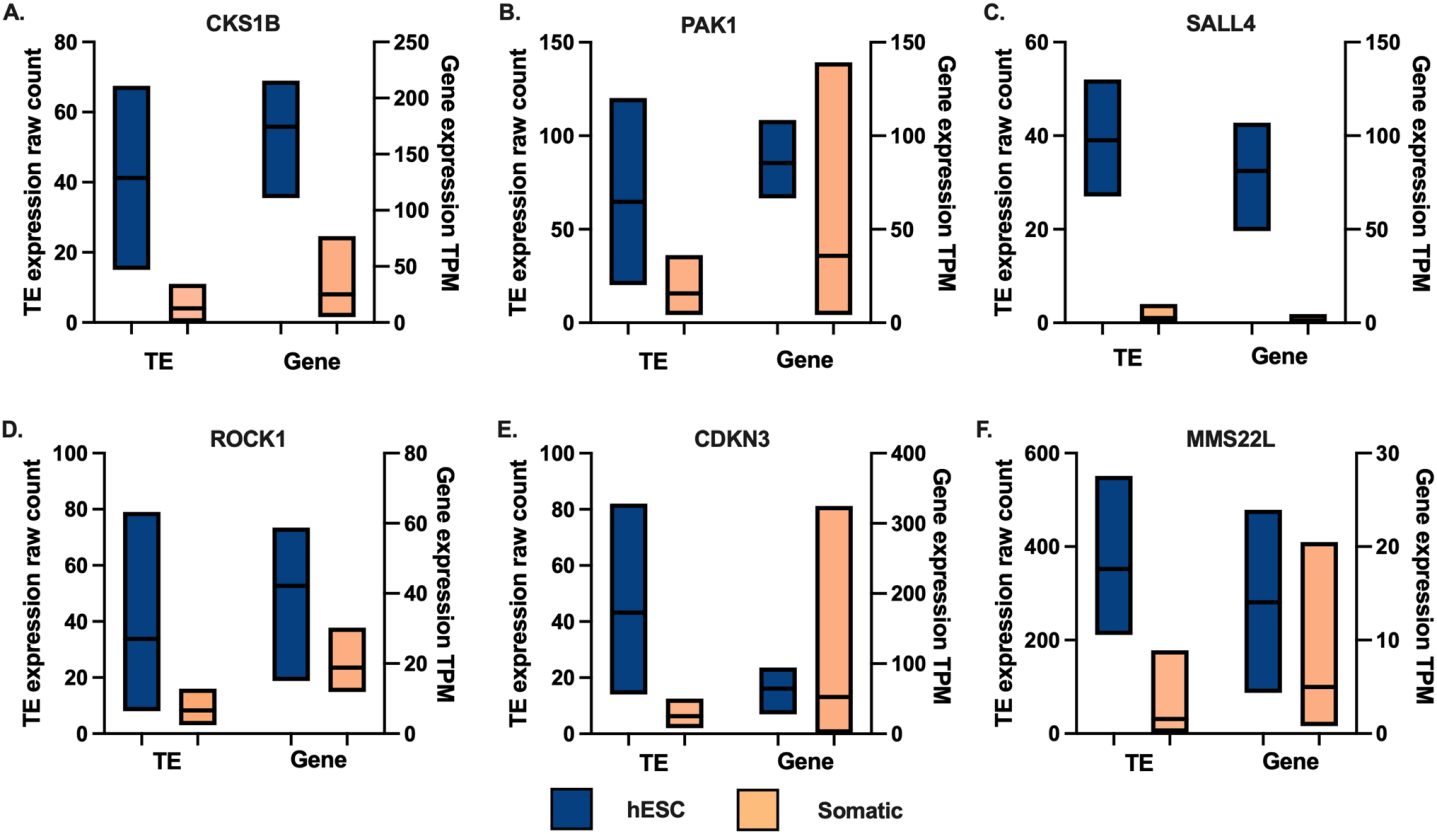
Expression of onco-exaptation candidates in hESC and somatic tissues. TE expression is plotted on the left *Y* axis and gene expression is plotted on the right axis. hESC *n* = 8, somatic *n* = 8 (brain, heart, kidney, liver, lung, ovary, testis and melanocyte)

Expression of onco-exaptation TEs was also investigated in the RNA-Seq data sets from hESCs. This analysis revealed that 35 TEs showed hESC enriched expression (raw count above 25 and fold change of more than 2 when compared to somatic). Of these, nine were among the 129 identified by Jang et al.as being tumour enriched. Expression of all candidates is provided in Supplementary Table 2. Expression of both the TE and corresponding oncogene in placenta and somatic tissue are plotted for six tumour enriched, hESC onco-exaptation candidates (Fig. 3). Both the TE and the oncogene show hESC enriched expression for *CDC28 Protein Kinase Regulatory Subunit 1B (CKS1B), Spalt-Like Transcription Factor 4 (SALL4)* and *Rho-Associated Coiled-Coil Containing Protein Kinase 1 (ROCK1)*, suggesting that the TE–oncogene regulatory relationship may have a hESC origin. The *P21 Activated Kinase 1 (PAK1), Cyclin Dependent Kinase Inhibitor 3 (CDKN3)* and *MMS22-Like DNA-Repair Protein (MMS22L)* genes are all expressed in somatic tissues but the TE expression is biased towards hESC, indicating that the TE may function to drive expression of the corresponding genes in a hESC-enriched manner (Fig. 3).

### RNA-Seq analysis of the ‘tumour-specific’ TE-derived transcript of LIN28B in placenta and somatic tissues

**Fig. 4:**
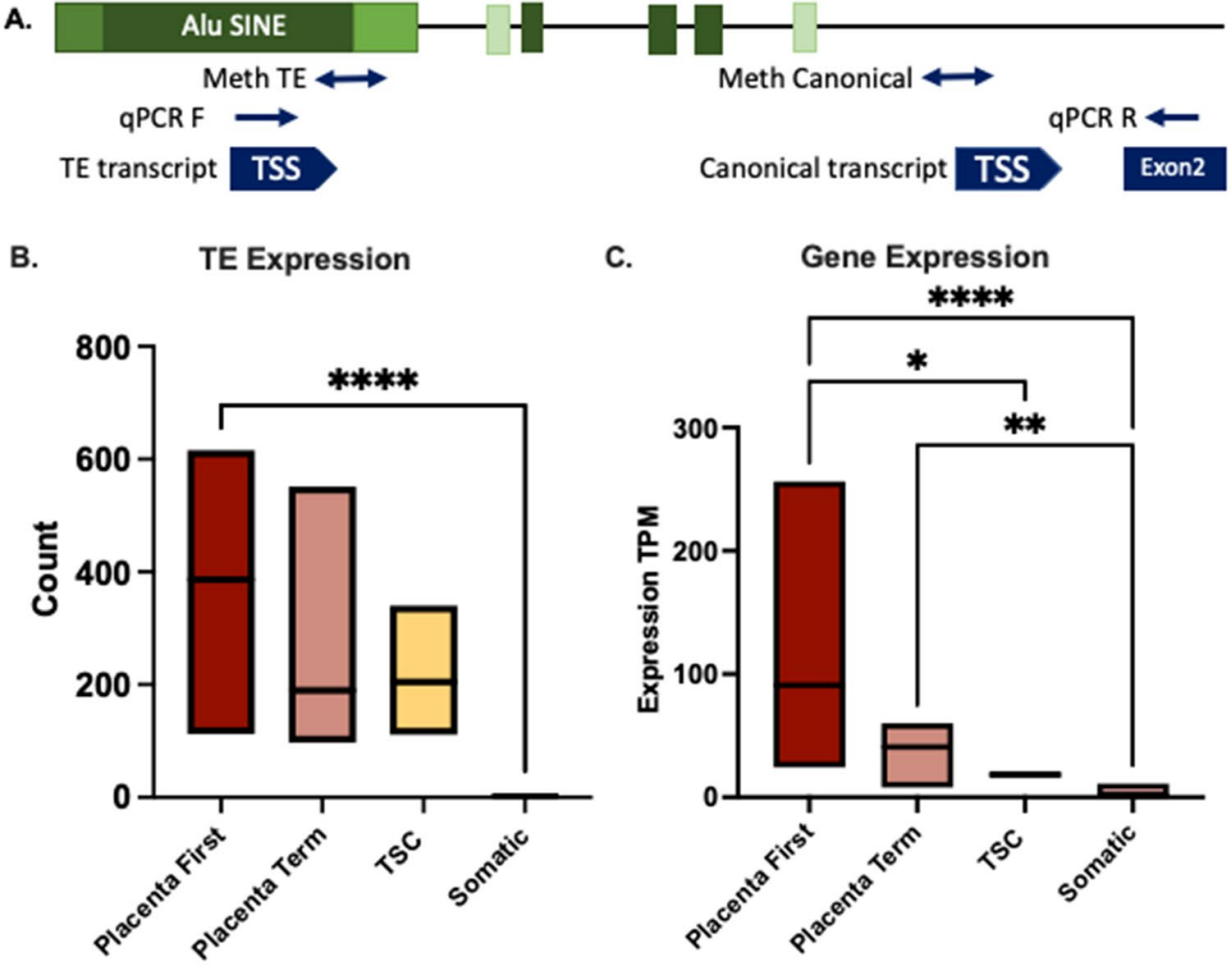
RNA-Seq expression quantification of LIN28B. **A** LIN28B locus with the AluSINE elements, amplicons for methylation analysis and rt-qPCR primers annotated. **B** Expression of the AluJbSINE/TE element that functions as an alternate promotor for LIN28B. Placenta First (First Trimester) *n* = 14, Placenta Term *n* = 8, TSC (trophoblast stem cell) *n* = 9, Somatic *n* = 8 (brain, heart, kidney, liver, lung, ovary, testis and melanocyte). ****P value < 0.0001(*T* test) B. Expression of LIN28B gene quantified by Stringtie. Placenta First Trimester *n* = 14, Placenta Term *n* = 8, Somatic *n* = 8 (brain, heart, kidney, liver, lung, ovary, testis and melanocyte). ****P value < 0.0001, ***P value 0.0001–0.001 (2 way ANOVA)

The *LIN28B*-TE-transcript was identified in the first instance by the onco-exaptation analysis. As this gene is widely studies and has established roles in both placental development and malignancy it was included in subsequent analysis. Subsequent analysis utilising the TE analysis pipeline, RepExpress (Stockwell et al. 2021) also identified this TE as one of the mostly highly expressed TEs in the placenta. Expression of the gene also quantified using a standard RNA-Seq pipeline (Fig. 4).

The TE that functions as an alternate promotor for *LIN28B* was quantified for expression using FeatureCounts. Expression was detected in all placental tissue samples (first Trimester and Term) and the trophoblast stem cell line but not in any of the eight healthy somatic tissues analysed. As shown in Fig. 4, LIN28B was expressed in both first trimester and term placental tissue samples, the trophoblast stem cell line but had low expression in the healthy somatic tissue samples. This is the first report of expression of the TE-transcript in a healthy human tissue sample and highlights the importance of exploring early developmental tissues before concluding that a transcript is tumour-specific.

### RT-qPCR validation of expression of the ‘tumour-specific’ TE-derived transcript of LIN28B

The limitations of RNA-Seq in the analysis of repetitive TE sequences are significant, due to the difficulty of aligning short reads derived from repetitive TEs to a reference genome accurately (Lanciano and Cristofari 2020). Therefore, to confirm expression of the ‘tumour-specific’ TE-derived transcript in placenta it is necessary to validate the expression of some candidate developmental-enriched TE-derived genes using reverse-transcriptase quantitative PCR (RT-qPCR). Primers to detect expression of the TE-derived transcript were used, these were designed by Guo et al. to study the TE-derived transcript of *LIN28B* that encodes a longer protein isoform with additional N-terminal amino acids (Guo et al. 2018).

**Fig. 5:**
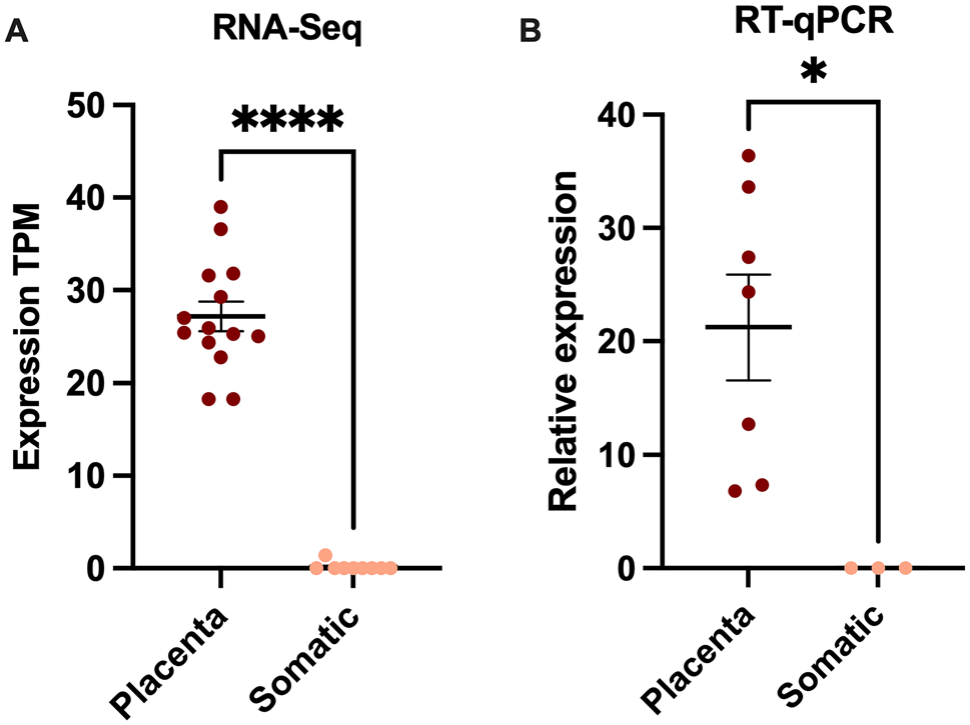
Quantification of expression of the TE-derived LIN28B transcript by RT-qPCR and RNA-Sequencing. **A** Expression of the TE-derived LIN28B transcript in first trimester placental tissues and somatic tissues quantified by RNA-Seq (placenta *n* = 14 (first trimester), somatic *n* = 9). **B** Expression of the TE-derived LIN28B transcript in first trimester placental tissue samples and somatic tissues as quantified by RT-qPCR. (placenta *n* = 7, somatic *n* = 3) ****P value < 0.0001, *P value 0.01–0.05 (Mann–Whitney test)

In both RT-qPCR and RNA-Seq analyses, expression of *LIN28B* was significantly upregulated in first trimester placental tissues compared to healthy somatic tissues (where no expression was observed) (Fig. 5).

### Targeted-deep bisulfite-sequencing of both the canonical and TE promotor of LIN28B

The gene expression analysis revealed that the “tumour-specific” TE-derived transcript of *LIN28B* is expressed in the placenta (in both first and third trimester placental tissues). DNA methylation is one of the most widely studied epigenetic mechanisms and plays a critical role in transcriptional regulation (Smith and Meissner 2013; Suzuki and Bird 2008). Importantly, DNA methylation at the promoter region of genes is strongly associated with transcriptional silencing (Lande-Diner et al. 2007). Furthermore, DNA methylation is largely considered to be involved in the long-term, stable silencing of gene expression (Siegfried and Simon 2010). DNA methylation has also been implicated in the repression of TE sequences (Jansz 2019). To this end, the majority of TEs are documented as being methylated in healthy somatic tissues (Friedli and Trono 2015; Hollister and Gaut 2009; Macaulay et al. 2017). However, loss of DNA methylation at TE sequences is a phenomenon that is well-characterised in both early developmental tissues and cancers (Altun et al. 2010; Ehrlich 2002; Novakovic and Saffery 2013; Schroeder et al. 2013). Previous studies have revealed that the SINE-derived alternate promotor for *LIN28B* is highly methylated in somatic tissues but is unmethylated in lung cancer cell lines (Jang et al. 2019), indicating that DNA methylation may be a mechanism for regulating expression of this transcript.

Here, CpG methylation of regions within both the TE-derived and canonical promoter of *LIN28B* was surveyed by targeted-deep bisulfite-sequencing (TDBS). Primers were designed to amplify a 228 bp region within the canonical TSS of *LIN28B*, this region contained six CpG sites. Additional primers were designed to target a 135 bp region within the SINE element-derived TSS to interrogate the methylation status of the 11 CpG sites within this amplicon. TDBS was carried out to determine whether the two promoters were differentially methylated between placental tissues and somatic tissues (blood and melanocyte).

**Fig. 6:**
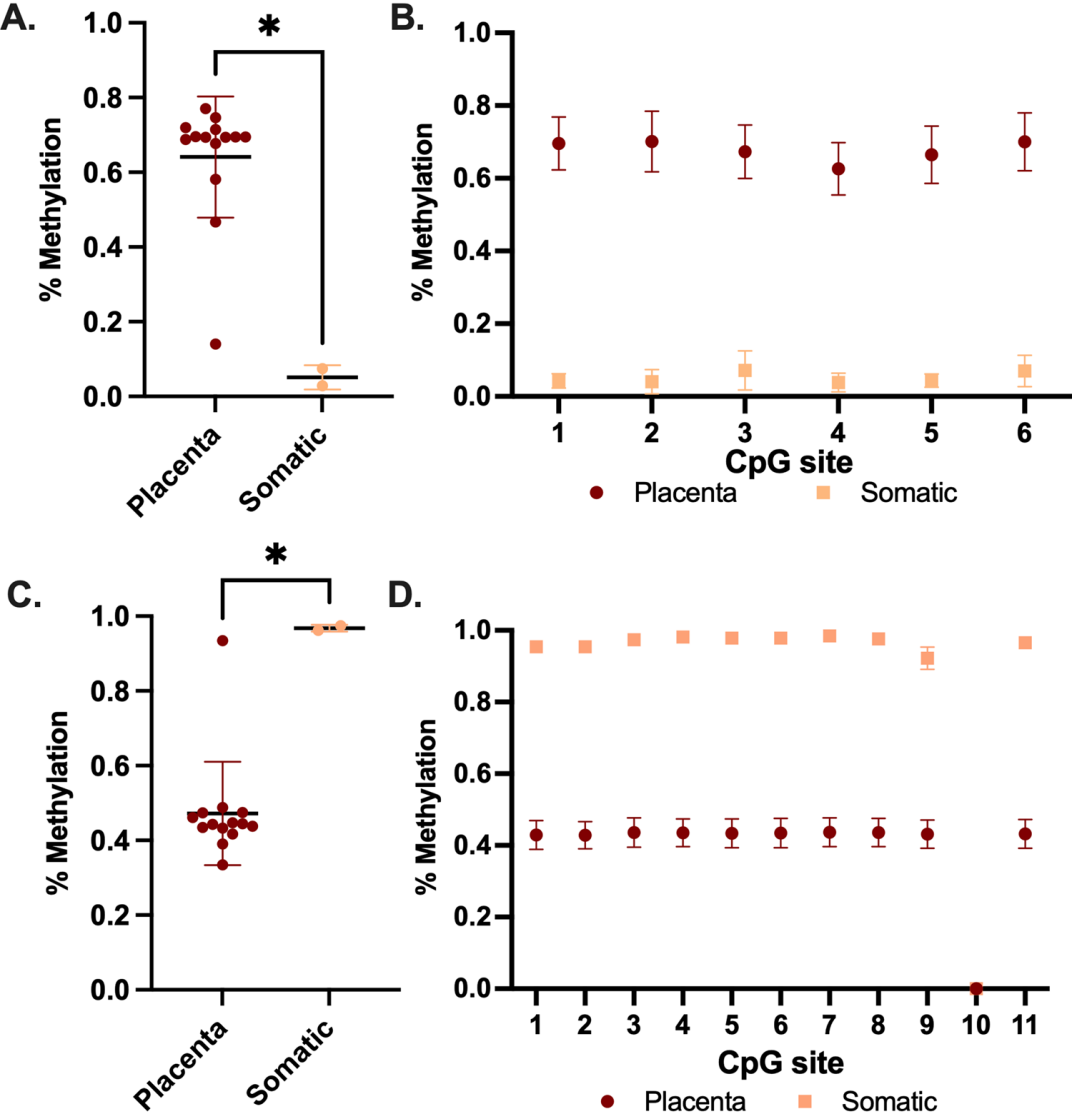
DNA methylation analysis results for LIN28B. **A** Mean CpG methylation for the canonical promoter of LIN28B (*P value = 0.0470—Kruskal–Wallis test) **B** Methylation of each CpG within the canonical LIN28B promoter amplicon. C. Mean CpG methylation for the TE-derived promoter of LIN28B (*P value = 0.0128—Kruskal–Wallis test). D. Methylation of each CpG within the TE-derived promoter of LIN28B. (Error bars represent SD, minimum read depth of 80). Placenta first trimester *n* = 14, somatic *n* = 2

TDBS on the two promoters for *LIN28B* revealed interesting results. The canonical promoter shows low mean promoter methylation levels in both healthy somatic tissue. In the placenta, the amplicon within the canonical promoter shows higher levels of methylation in most samples. There is a significant difference in mean promoter methylation of the somatic tissues (blood and melanocyte (p value = 0.047)) when compared to the placenta (Fig. 6A). There are low levels of variation in methylation between the six CpG sites surveyed within the canonical *LIN28B* promoter (Fig. 6B). Strikingly, the TE-derived promoter shows the inverse methylation pattern to the canonical. The mean promoter methylation for the TE amplicon is methylated at lower levels in placenta, and shows significantly higher methylation in both healthy somatic tissues (Fig. 6C). This result is interesting given that expression from both promoters occurs in the placenta. Importantly, the methylated status of the TE-derived promoter in somatic tissues confirms results from previous studies. Again, the methylation of all 11 CpG sites across the TE-derived promoter amplicon show a similar methylation pattern, although CpG ten gave no results for all samples surveyed (Fig. 6D).

## Discussion

Our analysis of onco-exaptation candidates in RNA-Seq data sets from early human developmental tissues provides convincing evidence to support reactivation of developmental TEs in cancer. Although there was not a clear majority of placental or hESC derived events, 26% tumour enriched events were expressed in either placental tissues or hESC data sets. This is further supported by the significant overlap between early developmental genes and oncogenes, suggesting that cancer cells can repurpose developmental pathways towards malignancy. Moreover, our results show for the first time that the tumour-specific, TE transcript of *LIN28B* is a placental transcript that becomes reactivated in tumours. *LIN28B* is a widely studied gene that has important roles in placental development and has also been shown to have a crucial role in driving malignancy. Our discovery that the TE transcript is expressed in the healthy human placenta, highlights the need for thorough investigation of a full complement of cell types, tissues and developmental stages before a transcript can be deemed tumour-specific. Moreover, it provides evidence for reactivation of developmental transcripts in tumours and the potential for these to drive malignant processes. However, the RNA-Seq data sets utilised for the onco-exaptation analyses were limited. The placental data sets were generated from tissue lysates and, therefore, do not necessarily represent all of the unique cell types of the placenta. The placenta is a heterogeneous tissue and the cell-type compositions differ between first trimester and term tissue lysates; therefore, it is likely that some information is lost when analysing tissue lysates rather than specific cell types. Furthermore, only hESC cell lines were analysed and the state of these cell lines was not well-characterised. Future work would benefit from assessing a greater range of hESC cell types and stages particularly by analysing them as they progress from a primed to pluripotent state. Although promising, it is possible that more developmentally expressed onco-exaptation events would be uncovered by analysing single-cell RNA-Seq data sets along with a greater range of developmental stages. Overall, this highlights the need for caution before concluding that transcripts are tissue-specific, particularly when concluding specificity to tumours.

Our results demonstrate the co-expression of TEs and oncogenes in a developmental-enriched manner and thus support the concept that some onco-exaptation events may not just arise spontaneously in cancer. Rather, they may occur as a result of dedifferentiation-associated epigenetic changes occurring in tumours, resulting in the reactivation of developmental TE–gene regulatory relationships. Furthermore, it is unknown whether dedifferentiation would facilitate the activation of placental transcripts in cancer. Dedifferentiation—the acquisition of an early developmental state—is a hallmark of cancer; however, the underlying mechanisms remain elusive. Some cancers reacquire an epigenetic landscape that is reflective of early development, which corresponds with increased phenotypic plasticity and facilitates the activation of early developmental genes. To this end, the fundamental regulators of pluripotency are all potent oncogenes, suggesting that cancers can repurpose developmental genes to drive malignancy. This idea is supported not only by the data presented here, but also by the extensive overlap between well-known oncogenes and early developmental genes, and the known role of TEs in regulating developmental genes in embryonic stem cells and the placenta (Sundaram and Wysocka 2020). Although referred to collectively here as early developmental tissues it is important to acknowledge the fundamental differences between embryonic stem cells and the placenta. It is known that cancer cells can acquire stem cell-like properties and reactivate genes associated with pluripotency. However, the mechanism for reactivation of placental genes in cancer is yet to be elucidated and could, therefore, be entirely distinct from that of pluripotency-associated genes. Moreover, it is not yet known whether some cancer stem cells are derived from the dedifferentiation of cancer cells or from stem cell populations that become malignant. Further work is needed to establish whether dedifferentiation may be a mechanism for the reactivation of placental genes in cancer and to further elucidate the functional implications of this.

Not all onco-exaptation events were found to have developmental expression. Further investigation of the remainder to determine whether they are novel to cancer may reveal the mechanisms by which onco-exaptation occurs. Furthermore, it is likely that analysing cell type and stage specific RNA-Seq data from early developmental tissues would reveal further onco-exaptation events that have a developmental origin. Current models for onco-exaptation do not acknowledge the potential for some events to be the result of reactivation of TE–oncogene regulatory relationships from early developmental tissues. The assumption that regulatory relationships between TEs and oncogenes are novel to cancer is often based solely on the absence of either in the corresponding somatic tissue. To the best of our knowledge, this was the first study to profile onco-exaptation events in either hESCs or in the placenta to evaluate whether they may have developmental origins.

The results presented in this study highlight the need for further work in this field. Specifically, including cell type specific and stage specific RNA-Seq data sets may reveal further onco-exaptation events that have a developmental origin. Furthermore, it would be important to validate expression using long-read sequencing technologies and targeted approaches. Traditional short read RNA-Seq analysis is limited in transcript assembly and analysis of TE sequences due to their repetitive nature. The analysis of RNA-Seq data presented here and the subsequent validation by RT-qPCR confirms transcription from the TE-derived transcript of *LIN28B* in the placenta. However, the methylation analysis of both *LIN28B* promotors indicate the need for further analysis of the regulatory mechanism that modulates promotor activity and subsequent expression. Guo et al. demonstrated that the TE/tumour transcript of *LIN28B* is regulated by Nuclear Transcription Factor Y Subunit Alpha (NFYA), while the canonical transcript is regulated by c-MYC in hepatocellular carcinoma cells. It would be particularly interesting to further investigate the mechanism that drives loss of DNA methylation, and further explore the mechanism for expression of the canonical transcript in placental cells despite the moderate levels of DNA methylation. The canonical promotor shows low levels of DNA methylation in the two somatic tissues analysed despite the lack of expression suggesting that DNA methylation is not the only mechanism for regulating *LIN28B* expression. Exploring the functions of each transcript in both the placenta and tumour cells is also an exciting prospect, Guo et al. state that the TE transcript is critical for cell proliferation and tumorigenesis in hepatocellular carcinoma and may present a new candidate for targeted therapy. However, no studies have explored the importance of this transcript in the placenta and whether it performs a distinct role to the canonical transcript. Moreover, it would be fascinating to explore when this divergent methylation and expression of both transcripts is established during embryonic development.

Promoter methylation did not show an inverse correlation with expression for either transcript of *LIN28B*. The canonical promoter showed higher methylation in placenta than in either somatic tissue, despite high expression of this transcript in the placenta. The TE promoter showed lower levels of methylation in placenta compared to somatic tissues, and the transcript expressed from this promoter was expressed in placenta. Intriguingly, despite lower levels of methylation at the TE-derived promoter in the placenta, the canonical transcript is still the predominantly expressed transcript in placenta. While the DNA methylation analysis was informative, the small regions of the promotor analysed, along with the nature of gene promotor regions, means that these result cannot be presumed to represent the entire promotor. It is possible that the region investigated in some assays was not in fact a critical part of the gene promoter and, therefore, may not reflect methylation levels across other regions. Furthermore, DNA methylation is only one epigenetic mechanism. It is now known that in many cases multiple epigenetic mechanisms work in combination to regulate gene expression (Vaissière et al. 2008). Although investigating DNA methylation changes at specific loci can provide some meaningful information on epigenetic regulation, it by no means imparts the entire story. Moreover, there is an increasingly nuanced relationship between DNA methylation and gene expression, highlighting the need for extensive investigation before drawing conclusions.

In conclusion, this study provides novel insights into the potential role of TEs in regulating gene expression during development and disease. Our findings demonstrate that some TE–oncogene interactions may not be cancer-specific but rather arise from the epigenetic reactivation of TE-derived regulatory events that are involved in early development. The identification of these TE-derived regulatory events in developmental tissues may provide new opportunities for the development of therapies that target TEs in diseases beyond cancer. Further studies are needed to explore the potential of these TE-derived regulatory events as therapeutic targets and to understand their molecular mechanisms. In addition, our findings suggest that the involvement of TEs in gene regulation during development may be more widespread than previously thought, highlighting the need for further studies to better understand the potential role of TEs in normal physiology.

## Methods

### Samples used for RNA-sequencing analysis

Due to limitations of the publicly available RNA-seq data from first trimester placental tissues, a cohort of 14 first trimester placental tissue lysates were sequenced. Data was generated for 8 healthy somatic control tissues (brain, heart, kidney, liver, lung, ovary, testis and melanocyte). A cohort of 16 publicly available term placental lysates and 7 hESC samples were obtained from the sequenced read archive (SRA—https://www.ncbi.nlm.nih.gov/sra) (Table 1).

**Table 1:** Data sets used for RNA-Seq analysis

Tissue	Samples	Data access	Paired-end + Stranded	Reads	Accession
Placenta first	14	Available on paper acceptance	Yes	125 bp	PRJNA952801
Somatic	8	Available on paper acceptance	Yes	125 bp	PRJNA952801
Placenta term	16	Publicly available	Yes	100 bp	GSE77085
hESC	8	1 generated 7 Publicly available	Yes	100 bp	GSE118106

### RNA-sequencing analysis

All data was aligned the reference genome GRCh38 using the Gencode v34 annotations. The RNA-Seq analysis pipeline involved adaptor trimming using the tool cleanadaptors from the DMAP package (Stockwell et al. 2014), genomic alignment was performed by STAR (Dobin et al. 2013). Transcript assembly and annotation was carried out with Stringtie to enable gene expression quantification (Pertea et al. 2015). Notably the alignment was optimised to allow for optimal recovery of TE-associated reads (–winAnchorMultimapNmax 100 and –outFilterMultimapNmax 100). For the onco-exaptation analysis two gene transfer format (GTF) files containing annotations for the TEs from onco-exaptation events identified by Jang et al. were created. One of these contained all onco-exaptation TEs and the other only the tumour enriched. Expression of the TEs was then quantified using FeatureCounts (Liao et al. 2014). Multimapping reads were assigned fractionally (M –fraction) and a minimum overlap of 25 bp was set (–minOverlap 25).

### Validation of RNA-sequencing data: RT-qPCR

The TE-derived transcript for *LIN28B* had already been investigated in cancer tissues. The primers from that study were used to validate expression of the TE transcript in placental tissues (Table 2) (Guo et al. 2018).

**Table 2:** Onco-exaptation candidates selected for validation by RT-qPCR

Gene name	Transcript	Primer name	Primer Sequence (5′–3′)	Product length	Exon
*LIN28B*	TE	F	TTA CAA GCA TGA GCC ACC G	181	TE-1
		R	GCT CTT CTC CAC CAC CTT TG		

Primers were ordered from Integrated DNA technologies (IDT), Singapore. On receiving the primers, they were eluted to 100 μM and then diluted to a 10 μM working stock with forward and reverse pooled.

Samples were selected based on the availability of RNA-Seq data and those that had been used for the targeted methylation assays (Table 3).

**Table 3:** Samples used for RT-qPCR validation experiments (Targeted-deep bisulfite-sequencing—TDBS)

Tissue	Samples	RNA-Seq	NanoString	TDBS
Placenta first trimester	7	Yes	Yes	Yes
Blood	1	No	Yes	Yes
Melanocyte	2	No	Yes	Yes

The mRNA starting material was diluted to give a total of 200 ng in 20 μL, which was further converted into complementary DNA (cDNA) via reverse transcription using the ThermoFisher high-capacity cDNA reverse transcription kit (Cat #4,368,814) following the manufacturer’s protocol. Starting material RNA was diluted to give a total of 200 ng in 20 μL for the PCR reaction. RT-qPCR reactions were set up with 1 × TaKaRa SYBR green master mix and *LIN28B* primers. Plates were ran on a LightCycler 2000 machine for 50 cycles. RT-qPCR data was analysed using the qBASE software package and a standard protocol to normalise expression to the three reference genes selected (*YWHAZ, TBP* and *PGK1*).

### Targeted-deep bisulfite-sequencing (TDBS) assays

To investigate the methylation status of the promoter regions of candidate genes, targeted methylation assays were carried out (Table 4). Primers were designed to the promoter region of both the canonical and TE-derived transcript for *LIN28B* (Table 5) using the MethPrimer tool created by the Li Lab at the Peking Union Medical College Hospital, Chinese Academy of Medical Sciences  (https://www.urogene.org/methprimer/). The first exon along with ~ 250 bp upstream was provided as input to the tool. The optimal length was set to 300 bp and primers were selected that had the maximum number of CpG sites along with between 40% and 65% GC content. Primers were checked for specificity using the BLAST tool (https://blast.ncbi.nlm.nih.gov/Blast.cgi). Illumina tags were added to the 5′ end of each primer to enable recognition for sequencing (Illumina forward tag: 5′ ACGACGCTCTTCCGATCT 3′ Illumina reverse tag: 5′ CGTGTGCTCTTCCGATCT 3′).

**Table 4:** Primers for TDBS assays

Gene name	Primer name	Primer Sequence (5′–3′)	Product length	CpGs
LIN28B	TSS-F	TTAGGGGGTTAGAAATTGGAGAG	228	6
	TSS-R	AAAATTCACAATAAAACAATAAAA		
	TE–F	AGTTTATTGGAATTTTTGGGTATTG	135	11
	TE-R	CACCCCAACCTAAACAAAATAATC

**Table 5:** Samples selected for TDBS assays

Tissue	Samples	RNA-Seq	qPCR
Placenta first	10	Yes	Yes
Placenta term	5	No	Yes
Blood	1	No	Yes
Melanocyte	1	No	Yes

Placental tissue lysates (first trimester and term) and somatic (blood and melanocyte) were selected for targeted deep bisulfite sequencing experiments (Table 5).

Genomic DNA was extracted bisulfite converted using the ZymoResearch EZ DNA Methylation, Direct Kit (Catalog #D5021). The manufacturer’s protocol was followed, with an input of 500 ng. Bisulfite DNA was eluted in 14 μL and stored at −20 °C.PCRs were performed using the KAPA HiFi HotStart Uracil + kit (Catalog #KK2802) from Roche. The reaction was carried out using a touchdown PCR protocol to reduce the time spent optimising for each primer pair. This involved an initial annealing temperature of 60 °C which was reduced by 0.5 °C per cycle for 20 cycles (Table 6).

**Table 6:** Touchdown PCR protocol used for TDBS assay first round PCR

	Step	Temperature	Time
1	Denature	98 °C	4 min
2	Denature	98 °C	30 s
3	Anneal	60 °C	30 s
4	Extend	72 °C	1 min
Repeat step 2–4 20×			
5	Denature	94 °C	30 s
6	Anneal	50 °C	30 s
7	Extend	72 °C	1 min
Repeat step 2–4 40×			
8	Extend	72 °C	5 min
9	Hold	12 °C	Infinite

Samples were pooled to contain all amplicons for each sample. Pooling was performed based on the strength of the band on the gel to try and ensure equal coverage for each amplicon. Following pooling each sample was cleaned using llumina TruSeq DNA Nano Beads at a 1:1 ratio to remove primer dimer and PCR reagents. To distinguish each sample uniquely after sequencing, a unique set of Illumina indices were added to each purified sample. These specific PCR primers amplified each amplicon within a sample creating a unique tag that was specific to the sample. The second round PCR with Illumina indices was performed with the protocol (Table 7).

**Table 7:** PCR protocol used for TDBS assay second round PCR

	Step	Temperature	Time
1	Denature	95 °C	2 min
2	Denature	98 °C	20 s
3	Anneal	60 °C	20 s
4	Extend	72 °C	20 s
Repeat step 2–4 10×			
5	Extend	72 °C	40 s
6	Hold	4 °C	Infinite

Samples were pooled based on the strength of the band on the gel, and recleaned with the llumina TruSeq DNA Nano Beads. Concentration of DNA libraries was then determined with the Qubit 1 × dsDNA HS Assay Kit. Libraries were diluted to 1 ng/μL and run on an Aligent Bioanalyzer high sensitivity DNA chip to assess fragment size and quality. Sequencing was performed on the iSeq 100 system.

Raw fastq files for each read were merged using PEAR. Following merging of files TrimGalore was used to remove adaptor sequences and low quality reads. Read quality was assessed using fastQC and poor qualify reads were excluded. Each amplicon sequence was pulled out using the first 10 bp of the forward primer. Individual samples were identified using the unique Illumina indices for each. Reads were then aligned to target regions using Biq_Analyzer. Alignments were then used to calculate methylation scores. This generated individual CpG and mean CpG methylation values for each amplicon and each sample.

### Statistical analyses

Statistical analysis were done in PRISM 8. To determine whether individual genes were differentially expressed between somatic and early developmental tissues (placenta or hESC), a Mann–Whitney test was used for non-normally distributed data and a Welch’s T test was used for normally distributed data. Normality of data sets was determined with a D’Agostino and Pearson test. To assess differential expression between more than two data sets that were not normally distributed, a Kruskal–Wallis test with Dunn’s multiple comparisons was performed (nonparametric). To determine differential expression between more than two data sets that were normally distributed, a Brown–Forsythe and Welch ANOVA test was performed.

## Supplementary Information

Below is the link to the electronic supplementary material.

Supplementary file1 (DOCX 160 KB)

## Data Availability

The term placental data sets and hESC data sets are available on SRA. The first trimester data will be submitted to SRA on acceptance of the paper.
