# Supplementary material for: The transposable element-derived transcript of LIN28B has a placental origin and is not specific to tumours
Source: Mol Genet Genomics. 2023 Jun 3;298(5):1045–58. doi: 10.1007/s00438-023-02033-1 (PMC10363060; doi:10.1007/s00438-023-02033-1)
Supplement: Supplementary file 1 — Supplementary file1 (DOCX 160 KB) [file 438_2023_2033_MOESM1_ESM.docx]

## Supplementary Information

Supplementary Table 1: Placental enriched onco-exaptation candidates.

| Gene Name | Chr | TE start | TE end | Strand | First trimester | Term | Placenta | somatic | FC |
| --- | --- | --- | --- | --- | --- | --- | --- | --- | --- |
| LIN28B | chr6 | 104936824 | 104937119 | + | 443.231 | 189.56 | 316.39 | 0.88 | 361.59 |
| MYCN | chr2 | 15908516 | 15908879 | + | 88.500 | 8.78 | 48.64 | 0.25 | 194.56 |
| STRA6 | chr15 | 74193228 | 74193363 | - | 15.462 | 54.78 | 35.12 | 0.88 | 40.14 |
| XCL1 | chr1 | 168559710 | 168561212 | + | 72.154 | 147.56 | 109.85 | 3.00 | 36.62 |
| ARID3A | chr19 | 958902 | 959139 | + | 26.692 | 51.22 | 38.96 | 1.50 | 25.97 |
| KLF5 | chr13 | 73074812 | 73074924 | + | 14.385 | 38.00 | 26.19 | 2.25 | 11.64 |
| BCL2 | chr18 | 63273560 | 63273648 | - | 31.077 | 176.00 | 103.54 | 12.88 | 8.04 |
| BCL2 | chr18 | 63227886 | 63228321 | - | 41.308 | 239.22 | 140.26 | 22.50 | 6.23 |
| BANP | chr16 | 87987775 | 87987979 | + | 20.923 | 49.67 | 35.29 | 8.13 | 4.34 |
| TBC1D1 | chr4 | 38115087 | 38115401 | + | 11.000 | 42.22 | 26.61 | 6.38 | 4.17 |
| NCOA3 | chr20 | 47648476 | 47648748 | + | 61.538 | 71.33 | 66.44 | 16.75 | 3.97 |
| NEDD4 | chr15 | 55968859 | 55971342 | - | 22.538 | 42.00 | 32.27 | 8.38 | 3.85 |
| BRD4 | chr19 | 15328774 | 15329071 | - | 26.385 | 67.52 | 46.95 | 14.50 | 3.24 |
| RRAS2 | chr11 | 14337092 | 14337772 | - | 35.692 | 163.56 | 99.62 | 32.88 | 3.03 |
| MDM2 | chr12 | 68837360 | 68837655 | + | 13.385 | 38.89 | 26.14 | 11.88 | 2.20 |
| SKI | chr1 | 2277284 | 2277630 | + | 17.462 | 77.57 | 47.52 | 21.75 | 2.18 |

Chr = Chromosome, TE = transposable element, FC = Fold change.

Supplementary Table 2: Human embryonic stem cell (hESC) enriched onco-exaptation candidates.

| Gene Name | Chr | TE start | TE end | Strand | Somatic average | Average hESC | FC |
| --- | --- | --- | --- | --- | --- | --- | --- |
| MMS22L | chr6 | 97240926 | 97244374 | - | 61 | 187.5 | 3.07 |
| RRAS2 | chr11 | 14337092 | 14337772 | - | 32.875 | 86.8 | 2.64 |
| PAK1 | chr11 | 77356700 | 77356895 | - | 15.625 | 54.3 | 3.48 |
| CKS1B | chr1 | 154975847 | 154976087 | + | 4 | 39.6 | 9.90 |
| CDKN3 | chr14 | 54404878 | 54407977 | + | 11.0625 | 36.1 | 3.26 |
| ROCK1 | chr18 | 21016908 | 21017183 | - | 8.25 | 28.5 | 3.45 |
| CKS1B | chr1 | 154975388 | 154975559 | + | 5 | 28.1 | 5.62 |
| MRE11A | chr11 | 94452814 | 94453792 | - | 10.5 | 28.0 | 2.67 |

Chr = Chromosome, TE = transposable element, FC = Fold change.


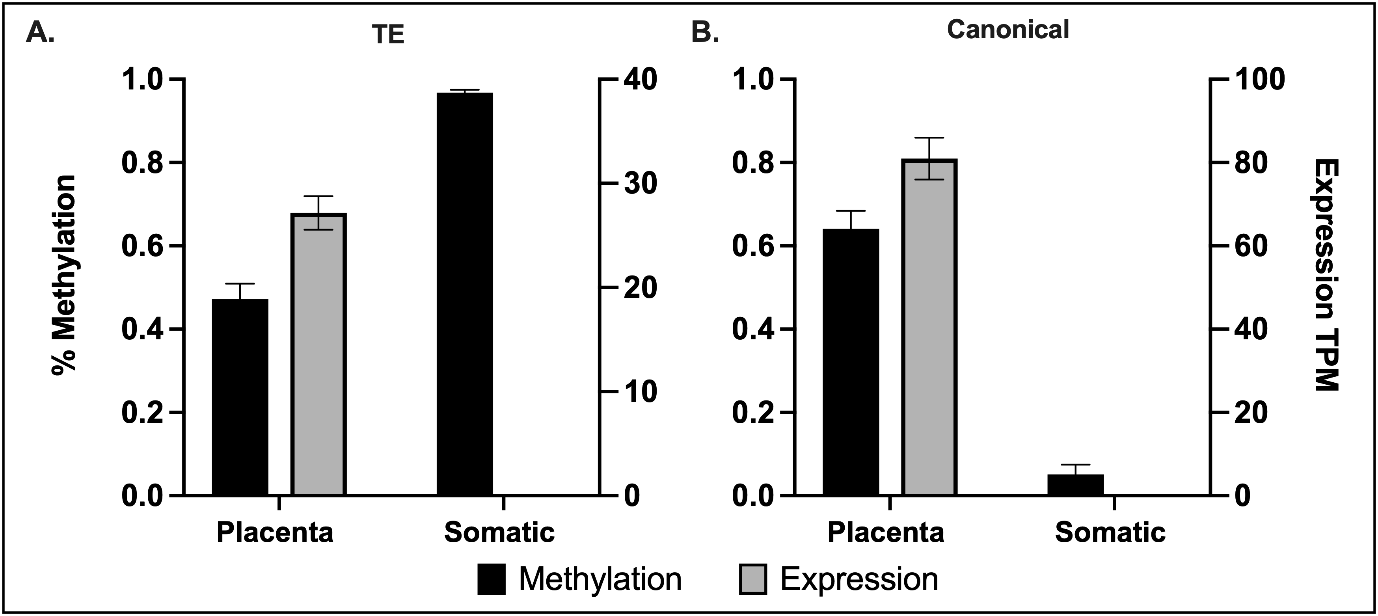


Supplementary Figure 1: Expression and Promoter Methylation for both transcripts of LIN28B. Methylation of the promoter region was determined by TDBS, and expression was quantified by RNA-sequencing. Mean promotor methylation (black bar and left Y-axis) and expression levels in TPM (grey bar and right Y-axis) of candidate genes in Placenta and Somatic Tissues. A. TE transcript B. Canonical Transcript. ). Placenta n=10, somatic n=3 (TDBS), Placenta n=33, somatic n=8 (RNA-Seq).
